# Assessment of Individual and Mixed Effects of Six Minerals on Thyroid Hormones in Chinese Pregnant Women

**DOI:** 10.3390/nu16030450

**Published:** 2024-02-03

**Authors:** Simeng Gu, Zhe Mo, Zhijian Chen, Xueqing Li, Yujie Jiang, Chenyang Liu, Fanjia Guo, Yahui Li, Guangming Mao, Xuemin Huang, Xiaofeng Wang

**Affiliations:** 1 Zhejiang Provincial Center for Disease Control and Prevention, Hangzhou 310051, China; smgu@cdc.zj.cn (S.G.); zhmo@cdc.zj.cn (Z.M.); zhjchen@cdc.zj.cn (Z.C.); xqli@cdc.zj.cn (X.L.); 2111101059@nbu.edu.cn (Y.J.); 22118893@zju.edu.cn (C.L.); gfjia@cdc.zj.cn (F.G.); 881012022129@hmc.edu.cn (Y.L.); gmmao@cdc.zj.cn (G.M.); xmhuang@cdc.zj.cn (X.H.); 2 Health Science Center, Ningbo University, Ningbo 315211, China; 3 School of Public Health, Zhejiang University, Hangzhou 310058, China; 4 School of Public Health, Hangzhou Medical College, Hangzhou 310053, China

**Keywords:** thyroid function, pregnancy, iodine, mineral

## Abstract

The biosynthesis of thyroid hormones is essential for brain and neurological development. It requires iodine as a key component but is also influenced by other nutrients. Evidence for the combined nutrient status in relation to thyroid hormones during pregnancy is limited. We aimed to investigate the joint associations of iodine, selenium, zinc, calcium, magnesium and iron with maternal thyroid functions in 489 pregnant women from Hangzhou, China. Serum levels of six essential minerals and thyroid function parameters were measured during the first antenatal visit. Linear regression, quantile g-computation and Bayesian kernel machine regression were used to explore the individual and joint relationships between the six minerals and thyroid hormones. Linear regression analyses revealed that calcium was positively associated with free triiodothyronine (FT3). Zinc was positively associated with free thyroxine (FT4). Iodine was negatively associated with thyroid-stimulating hormone (TSH) and positively associated with FT3 and FT4. The quantile g-computation and BKMR models indicated that the joint nutrient concentration was negatively associated with TSH and positively associated with FT3 and FT4. Among the six minerals, iodine contributed most to thyroid function. The findings suggested that maintaining the appropriate concentration of minerals, either as individuals or a mixture, is important for thyroid health during pregnancy.

## 1. Introduction

Thyroid hormones play fundamental roles in the growth and maturation of many tissues, especially the brain and skeleton. Pregnancy is a critical time window, because thyroid hormones are required for both maternal metabolism and embryo development [1]. During the first trimester of pregnancy, the fetus demand is totally dependent on thyroid hormone synthetized by the maternal thyroid. At this time, fetal brain development could be irreversibly affected by a lack of thyroid hormones [2,3]. Even though the fetal thyroid gland is almost functionally mature during late gestation, it has been shown that the transfer of thyroid hormones from the mother to the fetus is considerable [4]. Therefore, any minor change in maternal thyroid hormone homeostasis should be deserving of attention.

The thyroid hormones are susceptible to nutrition intake [5]. Several dietary nutrients make a concerted effort for thyroid function with optimum performance. Primarily, the intake of dietary iodine plays a prominent role in thyroid hormone biosynthesis. Thyroid hormones, namely thyroxine (T4) and triiodothyronine (T3), are characterized by the iodine atoms within their molecular structure [6,7]. When the physiological requirements of iodine are not met, multiple intrathyroidal autoregulatory changes lead to the increased synthesis of T3 at the expense of T4, even without an increase in circulating thyroid-stimulating hormone (TSH) [8]. Most notable, almost all T3 found in the developing cerebral cortex is derived through the local deiodination of T4. Although the mother could still maintain a euthyroid state, iodine deficiency in pregnancy is potentially damaging to the neurocognitive development of the fetus and neonate [9].

It is believed that proper functioning of the thyroid gland also requires a number of dietary components, such as zinc (Zn), selenium (Se), iron (Fe), calcium (Ca) and magnesium (Mg). Zn works as a co-enzyme factor for many enzymes that are involved in deiodinase enzymes activity, thyrotropin-releasing hormone (TRH) and TSH synthesis [10]. The human thyroid gland contains the highest Se content per gram of tissue. Se participates in the antioxidant defense in the thyroid by removing oxygen free radicals generated during the production of thyroid hormones [11]. Fe deficiency reduces the heme-dependent thyroperoxidase activity and blunts the utilization of thyroid hormones [12]. The role of Ca and Mg in thyroid hormone synthesis could be indirect. They act at the iodide-uptake step and the deiodination step [13,14]. The above pregnancy-related nutrition deficiencies are common in both developed and developing countries [15,16]. However, the extent to which the maternal nutrition status can impact thyroid function is not well understood.

Traditionally, toxicological or epidemiological studies have focused primarily on a single nutrient, without measuring or modifying the effects of other nutrients. Although such an approach may provide clues to the relationship existing between a particular nutrient and a specific disease, this cannot truly reflect the reflect real-world scenarios in which a daily diet for human contains a broad spectrum of nutrients. Both the absolute concentration and relative proportion of the nutrients affect the physiological function and health [17]. Then, more attention has been directed towards studying the joint effects of nutrition mixtures. For example, epidemiological studies have found that iodine and Se interact [18]. Animal studies have also found that Se protects the thyroid against peroxidative damage in the presence of iodine deficiency [19]. It is easy to find that these interactions are generally in two-way interaction models and may lead to highly unstable results when incorporating collinear variables [20]. It remains unclear how the interaction pattern and the efficacy of mixtures evolve with the varied levels of nutrient elements. In addition, the severe combined deficiency of both iodine and selenium has been shown to be associated with impaired mental and physical development [21]. Meanwhile, further study on the single or combined effects of mild-to-moderate mineral element deficiencies is limited.

Therefore, the aim of the present study was to explore the associations of six mineral elements with thyroid functions during pregnancy. Quantile g-computation and Bayesian kernel machine regression (BKMR) models were used to assess (1) the overall, single-exposure, and interactive health effects of the multi-nutrient mixtures, (2) the importance of each nutrient and (3) the exposure–response relationships.

## 2. Materials and Methods

### 2.1. Study Population

A cross-sectional study design was used in this study. Between March 2020 to October 2021, pregnant women who had their first antenatal examination from three hospitals of three major counties (Chunan, Qujiang and Putuo) in Zhejiang Province, China, were recruited. The inclusion criteria in this study were as follows: (1) 18 to 45 years of age; (2) the first antenatal visit was before 12 gestational weeks; (3) no family history of thyroid disease; (4) no endocrine diseases or chronic diseases, such as heart disease, hypertension, cancer and diabetes. Pregnant women who received injections of iodine-containing contrast media for medical imaging over the past year were excluded. In total, 489 eligible participants were included into this study.

After being notified of all study details, every pregnant woman signed an informed consent form. Then, a clinic-based face-to-face interview was given for data collection. At last, 5 mL whole blood samples were collected by trained nurses. Samples were transported to the hospital laboratory. The serum specimens were stored at −80 °C for further research. The study was conducted according to the guidelines of the Declaration of Helsinki and approved by the Ethics Committee of Zhejiang Provincial Center for Disease Control and Prevention.

### 2.2. Measurement of Serum Element Concentrations

The concentrations of Ca, Mg, Fe, Zn, Se and iodine were measured via inductively coupled plasma mass spectrometry (ICP-MS; Agilent 7900, Agilent Technologies, Santa Clara, CA, USA). A 0.1 mL serum specimen was added to a 1.9 mL diluent containing Triton X-100 (0.1%) and nitric acid (1.0%). Quality control measures were implemented for each 20-sample batch. The quality control measures included a blank sample, duplicate sample, standard reference material and quality control sample (standard lyophilized human serum reference material, Trace Elements Serum L-2, Seronorm, Billingstad, Norway); 90–110% of these elements were recovered in the standard reference material, and the quality control results were within the confidence intervals. The limit of detection was 0.25 μg/L for these 6 elements, and no sample was below the limit.

### 2.3. Maternal Thyroid Hormone Measurement

The measurement of serum thyroid hormones, including TSH, FT3 and FT4, was performed by using an electrochemiluminescence immunoassay with a Cobas Elecsys 411 (Roche Diagnostics, Mannheim, Germany), coupled with corresponding calibration materials, reagents and quality controls. The quality control procedure was carried out according to the manufacturer’s instructions and standards. To ensure the reliability of the results, all samples were analyzed after testing the quality control samples. The limits of detection (LODs) for TSH, FT3 and FT4 were 0.005 mIU/L, 0.6 pmol/L and 0.5 pmol/L, respectively. Among the studied serum samples, the detection rates of FT3 and FT4 were 100%. Meanwhile, the TSH concentrations of 7 samples were below the LOD. All of these observations below the LOD were substituted by LOD/2.

### 2.4. Statistical Analysis

The data distribution was summarized as the mean ± standard deviation (SD), median, percentiles and range for continuous variables and frequency for categorical variables. Spearman correlation analyses were conducted to evaluate the bivariate associations between the mineral elements. Since the concentrations of these elements and thyroid hormones were skewed, the data were log-transformed to achieve linearity in the model.

General linear regression. First, we assessed the associations between individual nutrients and thyroid hormones by comparing the second, the third and fourth quartiles to the first quantile of a nutrient ‘s concentration using linear regression models. Linear regression with a test for trends in the quantile process was also conducted. All multiple regression models included age, gestational week at sample collection, pre-pregnancy BMI, ethnic groups, education, occupation, passive smoking status and alcohol intake as covariates.

Quantile g-computation. Subsequently, we utilized quantile-based g-computation to assess the overall effect of the six nutrients on thyroid function and the contribution of each nutrient. Unlike the weighted quantile sum regression, the quantile g-computation model is a novel method that can be used to explore the effects of an exposure mixture; it does not require that all exposures have effects in the same direction [22]. In this model, we concerted the exposures into quartiles and fitted the linear models. Effect estimates represented changes in the outcome when all exposures were increased by one quartile. This model provided the weights of the individual components of the mixture to the overall estimate. The model was adjusted for age, gestational week at sample collection, pre-pregnancy BMI, ethnic groups, education, occupation, passive smoking status and alcohol intake.

Bayesian kernel machine regression. We found that the serum concentrations of some nutrients were highly correlated. Therefore, the associations of all nutrients combined and potential interactions between these nutrients and thyroid hormones were explored using BKMR models. BKMR could flexibly model the exposure mixture components that allow for non-linear associations and non-additive interactions among exposures [23]. The key feature of BKMR is that the exposure—response function does not require a priori specification. A kernel function in the equation accommodates the nonlinearity and the interaction between different components in the mixtures. In this study, we selected the Gaussian kernel function according to the reference manual. The model ran up to 50,000 iterations using the Markov chain Monte Carlo algorithm. Posterior inclusion probabilities (PIPs) were calculated to identify the relative importance of each nutrient to the joint mixture effect. The fitted model could provide summary statistics, including (a) the overall effect of nutrient mixtures on the differences in thyroid hormones, when all of predictors are at their 10th to 90th percentile, as compared to when all of them are at their 50th percentile; (b) the univariate associations between each nutrient and the thyroid hormone parameters, when all of nutrients were fixed at the 50th percentile; and (c) the potential single-nutrient interaction when each nutrient increased from its 25th to its 75th percentile, while the remaining nutrients were fixed at their 25th, 50th or 75th percentile.

All statistical analyses were performed using R software (Version 3.3.2). *p* values < 0.05 were considered statistically significant.

## 3. Results

### 3.1. Population Characteristics

Of the 489 pregnant women in this study, the mean age was 28.90 ± 4.83 years, with a pre-pregnancy BMI of 21.48 ± 3.11 kg/m^2^. The mean gestation weeks were 9.47 ± 1.34 weeks. The majority of the women were of Han ethnicity (97.55%). Nearly half of them were office workers (52.76%) and had bachelor degrees or higher (48.26%). A few of the women (18.81%) were exposed to passive smoking, and only 5.52% were alcohol users (Table 1).

### 3.2. Nutrient Concentrations during the First Trimester of Pregnancy

Table 2 presents the serum levels of mineral elements during the first trimester of pregnancy. All six mineral elements were detectable in the whole study population. The medians of serum Ca and Mg were 107.90 (range: 37.70–154.90) mg/L and 20.80 (range: 13.40–38.40) mg/L. The medians of serum Fe, Zn, Se and iodine were 1535.00 (range: 249.00–4941.00) μg/L, 888.00 (range: 413.00–2599.00) μg/L, 94.20 (range: 32.10–191.40) μg/L and 84.20 (range: 33.10–165.20) μg/L, respectively.

Figure S1 shows the correlations between these nutritional elements. Maternal serum Ca was highly correlated with Mg (r = 0.72, *p* < 0.001), and modest correlations were observed among Fe, Zn and Se, with correlation coefficients ranging from 0.33 to 0.48. Additionally, iodine did not show any strong correlation with other serum elements.

### 3.3. Linear Regression Analyses

We first evaluated the associations between maternal nutritional elements and thyroid hormones using linear regression models (Table S1). After adjusting for covariates, the Ca concentration was positively associated with FT3 (*p* for trend = 0.026). The Zn concentration was positively associated with FT4 (*p* for trend = 0.004). The iodine concentration was negatively associated with TSH and positively associated with FT3 and FT4 (all *p* for trend < 0.001). Estimated associations for the quartile-specific concentrations of Mg, Fe and Se did not suggest obviously positive or negative trends.

### 3.4. Quantile g-Computation

Table 3 presents the quantile g-computation estimates for the overall effects of all nutrients on thyroid function. The nutrient mixture was found to be negatively associated with TSH (β = −0.43, 95%CI = −0.60, −0.25, *p* < 0.001) and positively associated with FT3 (β = 0.06, 95%CI = 0.04, 0.08, *p* < 0.001) and FT4 (β = 0.08, 95%CI = 0.06, 0.10, *p* < 0.001).

As displayed in Figure S2 and Table S2, serum iodine, Fe and Ca accounted for the negative weights for the associations of the nutrient mixture with TSH (iodine = −0.75, Fe = −0.17 and Ca = −0.08), while Se, Zn and Mg contributed to the positive weights (Se = 0.50, Zn = 0.45 and Ca = 0.06). For both FT3 and FT4, Se and Mg were negatively weighted, and iodine, Ca, Zn and Fe were positively weighted. Among the nutrients, iodine explained the largest proportion of the overall effects on thyroid function.

### 3.5. BKMR

The PIPs derived from BKMR models are shown in Table S3. PIP is a ranking measure to explain the extent to which data favors the inclusion of a variable in the regression. Undoubtedly, iodine contributed most to the joint effect of the nutrient mixture on all hormones (PIPs = 1.00). In addition, Ca had high PIPs for TSH and FT4 (PIP = 0.49 and 0.58, respectively). The PIP of selenium was high for FT4 (PIP = 0.67).

**Figure 1:**
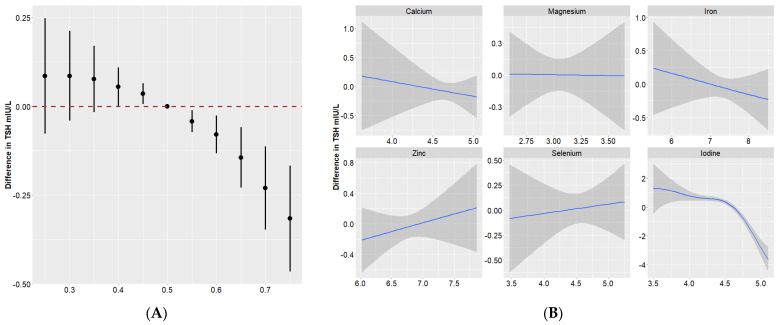
Overall and individual effects of minerals on TSH in Bayesian kernel machine regression. (**A**) Overall effect (95% Credible Interval) of all minerals at a particular percentile compared with their 50th percentile. (**B**) Single effect of each mineral on TSH when all of the other minerals were fixed as the 50th percentile. The blue solid lines and the gray bands represent the coefficient and its 95% confidence intervals.

**Figure 2:**
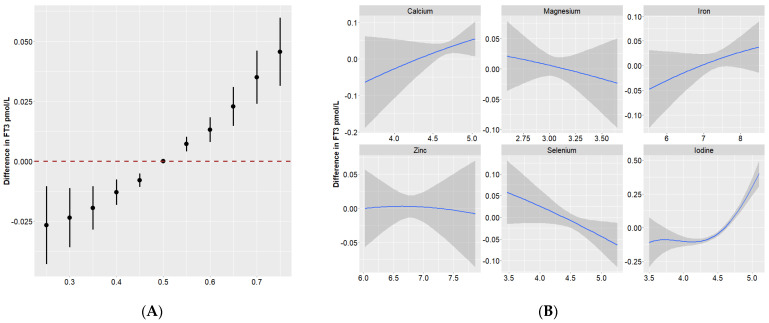
Overall and individual effects of minerals on FT3 in Bayesian kernel machine regression. (**A**) Overall effect (95% Credible Interval) of all minerals at a particular percentile compared with their 50th percentile. (**B**) Single effect of each mineral on FT3 when all of the other minerals were fixed as the 50th percentile. The blue solid lines and the gray bands represent the coefficient and its 95% confidence intervals.

**Figure 3:**
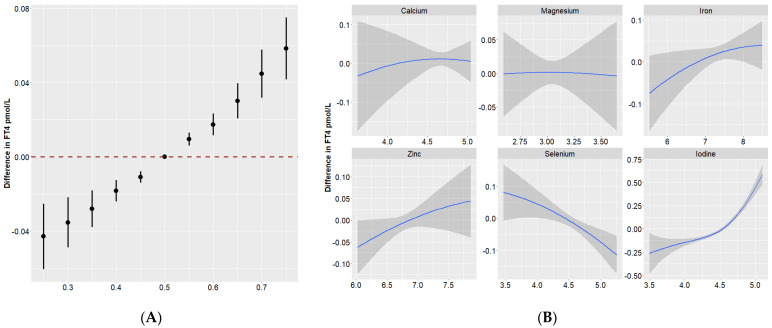
Overall and individual effects of minerals on FT4 in Bayesian kernel machine regression. (**A**) Overall effect (95% Credible Interval) of all minerals at a particular percentile compared with their 50th percentile. (**B**) Single effect of each mineral on FT4 when all of the other minerals were fixed as the 50th percentile. The blue solid lines and the gray bands represent the coefficient and its 95% confidence intervals.

Figure 1A illustrates the dose–response relationship of the overall effect of the nutrient mixture and the effect of individual nutrients on maternal thyroid hormones. Compared to the 50th percentile, serum TSH showed a significant decrease when all of these nutrients were at their 55th percentile or above. There were positive trends of nutrients in relation to FT3 and FT4 (Figure 2A and Figure 3A).

We also estimated the univariate summaries of the changes in thyroid hormones associated with a change in a single nutrient concentration from its 25th to 75th percentile, while all the other nutrients were fixed at a particular threshold (25th, 50th or 75th percentile). We found that iodine was the only one presenting a significant effect on all hormones. Se was negatively associated with FT3 and FT4. And these associations seemed to be stronger when other nutrients were at their higher percentiles (Figure S3).

To further investigate the potential nonlinearity of the exposure response function, we then assessed the univariate exposure response function between these nutrients and thyroid hormones (when all other nutrients were at a median level). Figure 1B, Figure 2B and Figure 3B suggested that dose–response relationships between iodine and thyroid function parameters were (reverse) L-shaped. Thyroid hormones generally leveled off when the serum iodine concentration was no more than 90 μg/L. The associations of Se with FT3, as well as FT4, were approximately linear.

## 4. Discussion

In this cross-sectional study, we evaluated the associations of multiple nutrients with thyroid hormone parameters during pregnancy using three statistical models. Our results showed that the nutrient mixture had a negative effect on TSH and had positive effects on FT3 and FT4. Moreover, significant associations of individual nutrient with thyroid function parameters were also seen. When comparing the results from different models, we found consistent associations between these nutrients and thyroid hormone parameters (Table S3). Our findings highlighted the importance of assessing the effect of a single and nutrient mixture on thyroid function.

### 4.1. Effects of Iodine on Thyroid Function

In this study, we found that iodine was the key contributor to thyroid hormones, being negatively associated with TSH. The increase in TSH is due to the depletion of iodine stores. The consequence of this would be the elevated TSH concentration and relative hypothyroxinemia [24]. Moreover, serum iodine was positively associated with FT3 and FT4, which is consistent with the previous findings from Pan et al. [25]. The correlations between serum iodine and thyroid hormones were much stronger than correlations between urinary iodine and thyroid hormones. The results indicated that serum iodine is closely correlated with the bioavailable iodine supply in the thyroid and provide more information than urinary iodine [26].

### 4.2. Effects of Se on Thyroid Function

We found that there were inverse associations between Se and FT3, as well as FT4, which is consistent with the findings from previous epidemiological studies. In a Korean study, serum Se levels were positively correlated with FT4 in women [27]. Se is required for the expression of the selenoenzymes deiodinase type I–III. Type I and II deiodinases (D1 and D2) activate T4 to T3, whereas Type III deiodinase is the major inactivating enzyme of both T4 and T3 [28,29]. It was hypothesized that the activity of deiodinase, becomes depressed with a high dietary Se intake. With excessive Se intake, impaired regulation of the thyroid hormone axis could be observed with typical decreases in serum T3.

### 4.3. Effects of Zn on Thyroid Function

We found that the first-trimester serum Zn was positively associated with FT4, but not with TSH, which is consistent with previous findings from Dutch pregnant women [30]. The thyroid hormones increasing with Zn might be due to zinc’s role in the metabolism of thyroid hormones. Zinc is involved in regulating the activity of deiodinase enzymes, which contribute to the synthesis and body concentration of the T3 and T4 hormones [31]. Further, Zn could influence iodine utilization in the thyroid. Zn supplementation would increase T3 and T4 concentrations [32]. In addition, Zinc is also a structural component of the T3 receptors. However, some studies still failed to confirm the relationship between zinc and thyroid activity. A recent systematic review provided inconclusive evidence with respect to the association between the zinc concentration and thyroid hormone levels due to the divergence of research findings [33].

### 4.4. Effects of Ca and Mg on Thyroid Function

Current data concerning the effects of Ca on thyroid function are very limited. We found that there were positive associations between serum calcium and FT3, as well as FT4. Which is in accordance with the study performed by Mani et al. [34], in contrast to the study performed by Athokpham et al. [35], where they have shown no correlation between serum Ca and FT4. Thyroid hormones act directly on bone resorption, thereby maintaining calcium homeostasis. Both FT3 and FT4 exert a stimulating effect on osteoblastic differentiation in the bones, which would promote bone resorption and further increase blood Ca levels.

Experimental data have revealed that the Mg status could affect thyroid activity [36]. It acted at the iodide uptake step and deiodination during thyroid hormone synthesis. We did not find a significant association between Mg and thyroid function biomarkers, and the nutrient mixture model did not show that Mg makes a great contribution to the overall effect, which is consistent with the findings from previous studies. Cross-sectional studies found no significant association between Mg and thyroid hormones [37]. Only a severely low serum Mg level is associated with an increased risk of hypothyroidism [38]. However, it is well known that the Mg level could be related to thyroid cancer. A case-control study also revealed that a low serum Mg concertation combined with reduced urinary iodine is associated with an increased risk of thyroid goiter [39]. These data supported the association between Mg and thyroid function.

### 4.5. Effects of Fe on Thyroid Function

Fe is an important component for the production and transformation of thyroid hormone. Fe deficiency could reduce TPO activity, damage peripheral T4–T3 conversion, and stimulate TSH secretion [40]. In this current study, we did not observe any significant association between Fe and thyroid hormones. Nevertheless, we found that a higher Fe concentration showed a trend towards lower TSH and higher FT3 and FT4 concentrations during the first trimester of pregnancy, which is consistent with the previous findings. Findings from both epidemiological and experimental studies has demonstrated that a lack of Fe could break the thyroid hormone metabolism. Animal studies showed that rats fed Fe-deficient diets have significantly lower T3 and T4 compared to rats fed adequate Fe [41]. Studies in pregnant women also showed that, in comparison with healthy controls, Fe deficiency was accompanied by reduced serum FT4 and FT3 [42,43]. Compared to previous studies, we thought that the reason for the weak associations between Fe and thyroid hormones might be that the study population that we included comprised healthy pregnant women. They do not have a severe Fe deficiency, such as Fe deficiency anemia. This makes it difficult to evaluate how severe low iron stores affect the thyroid functions of pregnant women.

There are several limitations to this study. First, this is a cross-sectional study. It is not possible to discern causal relationships. Second, we did not evaluate the urinary iodine concentration in the studied sample. As a result, we could not classify the daily iodine intake according to the WHO classification. It may also be advisable to have data on the level of other minerals, such as copper. Copper has an important role in maintaining thyroid health. Copper could stimulate the production of T4 and prevent T4 overabsorption [44]. Furthermore, copper could be involved in the cytotoxic ROS effects in thyrocytes [45]. Animal studies found that relatively higher levels of copper were found to be associated with hypothyroidism, while relatively lower levels of copper were found to be associated with hyperthyroidism [46,47]. Jain [48] reported that for males, serum copper was associated with increased levels of FT4 and TT4; for females, serum copper was associated with increased levels of TT3 and TT4. Third, we did not measure the human chorionic gonadotropin (hCG) level, which is an important determinant of gestational thyroid function. Fourth, although our findings could be a reference about the associations of a nutrient mixture with thyroid function during the first trimester, we should stress that it might not be applicable to other populations (e.g., pregnant populations in the second or third trimesters). Fifth, some confounding factors, such as dietary habits, were unavailable, which might confine the representativeness of our findings. Moreover, nutrient requirements increase during pregnancy. It is necessary to determine whether or not an exposure threshold exists. Further studies are warranted to validate the above findings and elucidate the mechanisms underlying the effects of nutrient exposure on thyroid function.

## 5. Conclusions

In summary, our findings suggests that mineral elements, mostly iodine, influence thyroid function during pregnancy. When considering these elements as a mixture, the joint element status was negatively associated with TSH and positively with FT3 and FT4. Further studies on the potential implications for maternal and early childhood health are warranted.

## Figures and Tables

**Table 1:** Characteristics of pregnant women included in this analysis.

Characteristics	*N* = 489
Maternal age (year, mean ± SD)	28.90 ± 4.83
Gestational week at sample collection (weeks, mean ± SD)	9.47 ± 1.34
Ethnicity [*n* (%)]	
Han	477 (97.55)
Others	12 (2.45)
Pre-pregnancy BMI, [*n* (%)]	
<18.5	79 (16.16)
18.5–23.9	324 (66.26)
≥24.0	86 (17.59)
Education, [*n* (%)]	
Middle school or lower	116 (23.72)
High school	137 (28.02)
Bachelor degree or higher	236 (48.26)
Occupation, [*n* (%)]	
Office workers	258 (52.76)
Domestic workers	115 (23.52)
Others	116 (23.72)
Passive smoking exposure, [*n* (%)]	
No	397 (81.19)
Yes	92 (18.81)
Pre-pregnancy alcohol intake, [*n* (%)]	
No	462 (94.48)
Yes	27 (5.52)
Thyroid function biomarkers	
TSH (mIU/L)	1.11 (0.52, 1.78)
FT3 (pmol/L)	4.69 (4.33, 5.15)
FT4 (pmol/L)	16.37 (14.82, 17.90)

Note: SD, standard deviation; BMI, body mass index; TSH, thyroid stimulating hormone; FT3, free triiodothyronine; FT4, free thyroxine.

**Table 2:** Data distributions of the six mineral elements.

Element	Mean ± SD	P2.5	P25	P50	P75	P97.5	Range
Calcium (mg/L)	107.37 ± 13.15	78.58	101.90	107.90	114.80	131.28	37.70–154.90
Magnesium (mg/L)	21.08 ± 2.64	16.42	19.50	20.80	22.50	26.28	13.40–38.40
Iron (μg/L)	1571.1 ± 534.49	738.40	1264.00	1535.00	1807.00	2628.00	249.00–4941.00
Zinc (μg/L)	907.38 ± 240.48	553.80	777.00	888.00	989.00	1388.40	413.00–2599.00
Selenium (μg/L)	94.81 ± 21.58	50.88	81.10	94.20	106.40	139.92	32.10–191.40
Iodine (μg/L)	85.77 ± 20.29	54.00	71.50	84.20	95.80	132.80	33.10–165.20

**Table 3:** Quantile g-computation estimates for the joint effects of all nutrients on thyroid function.

Thyroid Function Parameters	β (95%CI)	*p*
TSH	−0.43 (−0.60, −0.25)	<0.001
FT3	0.06 (0.04, 0.08)	<0.001
FT4	0.08 (0.06, 0.10)	<0.001

Note: TSH, thyroid stimulating hormone; FT3, free triiodothyronine; FT4, free thyroxine.

## Data Availability

The data presented in this study are available on request from the corresponding author. The data are not publicly available due to policy.

## Supplementary Materials

The following supporting information can be downloaded at: https://www.mdpi.com/article/10.3390/nu16030450/s1, Figure S1: Spearman correlation coefficients across the studied mineral elements; Figure S2: Contribution weight of each nutrient to the overall effect on thyroid function parameters from the quantile g-computation model; Figure S3: Single-exposure effects of each individual nutrient on thyroid hormone parameters from BKMR; Table S1: Associations of maternal calcium, magnesium, iron, zinc, selenium and iodine concentrations with thyroid hormone levels in the general linear regression; Table S2: Contribution proportions of each nutrient to thyroid function parameters in the quantile g-computation; Table S3: Posterior inclusion probabilities (PIPs) of each nutrient in BKMR; Table S4: Directions of associations between single nutrient, multi-nutrient mixtures and thyroid function parameters in three statistical methods.
