# Supplementary material for: Assessment of Individual and Mixed Effects of Six Minerals on Thyroid Hormones in Chinese Pregnant Women
Source: Nutrients. 2024 Feb 3;16(3):450. doi: 10.3390/nu16030450 (PMC10857285; doi:10.3390/nu16030450)
Supplement: Supplementary file 1 [file nutrients-16-00450-s001.zip › nutrients-2824171-supplementary.pdf]

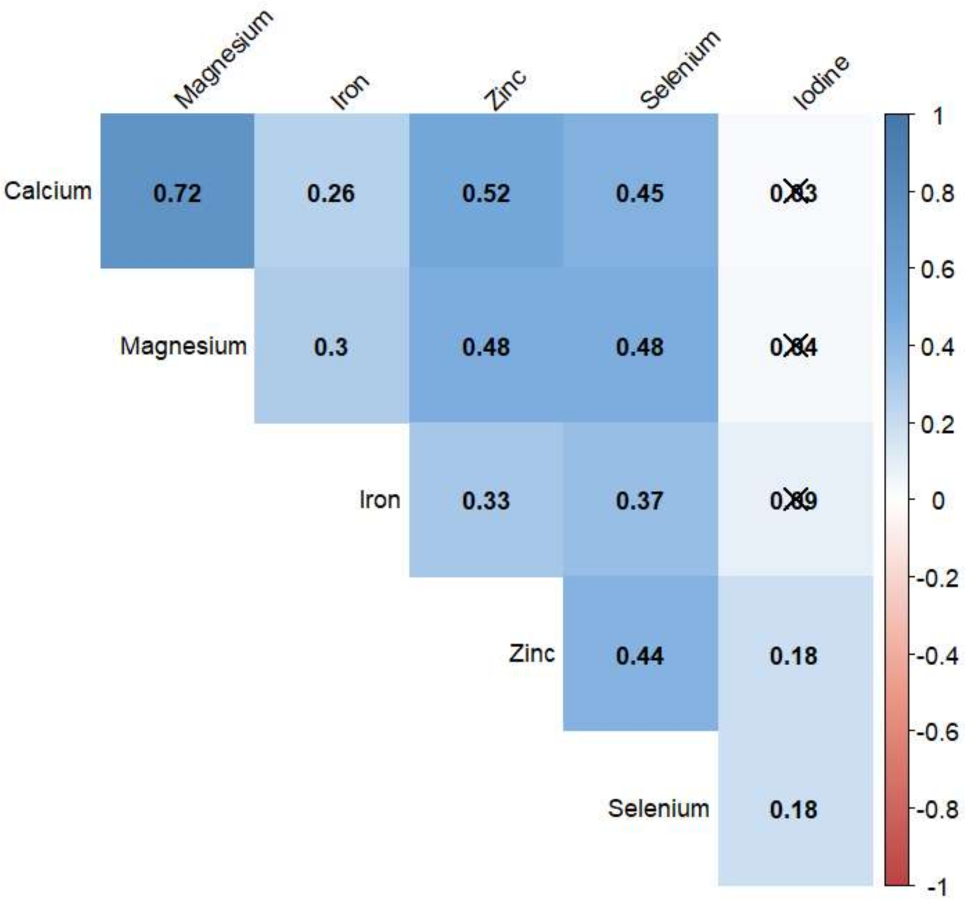

Figure S1. Spearman correlation coefficients across the studied mineral elements.

Table S1 Associations of maternal calcium, magnesium, iron, zinc, selenium and iodine concentrations with thyroid hormone levels in general linear regression

Element	Levels of concentration	TSH		FT3		FT4	
		β (95%CI)	*P*	β (95%CI)	*P*	β (95%CI)	*P*
Calcium (ng/L)	Quartile 1	Ref		Ref		Ref	
	Quartile 2	-0.29 (-0.65,0.07)	0.112	**0.05 (0.00,0.09)**	**0.032**	**0.07 (0.03,0.12)**	**0.002**
	Quartile 3	-0.25 (-0.60,0.11)	0.167	**0.05 (0.01,0.09)**	**0.024**	**0.05 (0.00,0.09)**	**0.046**
	Quartile 4	-0.22 (-0.59,0.14)	0.233	**0.05 (0.01,0.10)**	**0.020**	**0.06 (0.01,0.11)**	**0.017**
	P for trend		0.292		**0.026**		0.059
Magnesium (ng/L)	Quartile 1	Ref		Ref		Ref	
	Quartile 2	0.13 (-0.23,0.49)	0.479	0.00 (-0.04,0.05)	0.830	0.00 (-0.05,0.05)	0.982
	Quartile 3	-0.10 (-0.46,0.25)	0.577	0.02 (-0.02,0.06)	0.391	0.03 (-0.02,0.07)	0.248
	Quartile 4	-0.09 (-0.46,0.27)	0.617	0.01 (-0.03,0.05)	0.675	0.01 (-0.04,0.06)	0.710
	P for trend		0.392		0.552		0.485
Iron (μg/L)	Quartile 1	Ref		Ref		Ref	
	Quartile 2	0.00 (-0.36, 0.35)	0.986	0.01 (-0.04,0.05)	0.775	-0.01 (-0.06,0.03)	0.590
	Quartile 3	-0.26 (-0.62,0.10)	0.160	0.03 (-0.01,0.07)	0.188	0.04 (-0.01,0.08)	0.147
	Quartile 4	-0.19 (-0.55,0.16)	0.290	0.02 (-0.03,0.06)	0.479	0.01 (-0.03,0.06)	0.574
	P for trend		0.156		0.330		0.261
Zinc (μg/L)	Quartile 1	Ref		Ref		Ref	
	Quartile 2	-0.09 (-0.45,0.27)	0.621	0.04 (0.00,0.08)	0.081	0.03 (-0.02,0.07)	0.275
	Quartile 3	-0.13 (-0.48,0.23)	0.485	0.03 (-0.01,0.07)	0.156	0.03 (-0.02,0.08)	0.208
	Quartile 4	-0.12 (-0.48,0.23)	0.503	0.04 (0.00,0.08)	0.083	**0.07 (0.02,0.12)**	**0.003**
	P for trend		0.486		0.126		**0.004**

Selenium (μg/L)	Quartile 1	Ref		Ref		Ref	
	Quartile 2	-0.07 (-0.43,0.28)	0.690	0.02 (-0.02,0.06)	0.333	0.03 (-0.02,0.07)	0.293
	Quartile 3	-0.15 (-0.51,0.21)	0.403	0.00 (-0.05,0.04)	0.915	-0.01 (-0.05,0.04)	0.802
	Quartile 4	-0.18 (-0.53,0.18)	0.331	0.01 (-0.04,0.05)	0.730	0.01 (-0.04,0.06)	0.730
	P for trend		0.290		0.992		0.937
Iodine (μg/L)	Quartile 1	Ref		Ref		Ref	
	Quartile 2	-0.19 (-0.54,0.15)	0.266	0.02 (-0.02,0.06)	0.359	**0.06 (0.02,0.10)**	**0.006**
	Quartile 3	**-0.39 (-0.74,-0.04)**	**0.031**	**0.06 (0.01,0.10)**	**0.009**	**0.10 (0.06,0.15)**	**<0.001**
	Quartile 4	**-1.19 (-1.55,-0.84)**	**<0.001**	**0.16 (0.12,0.20)**	**<0.001**	**0.24 (0.19,0.28)**	**<0.001**
	P for trend		**<0.001**		**<0.001**		**<0.001**

Note: TSH, thyroid stimulating hormone; FT3, free triiodothyronine; FT4, free thyroxine. All thyroid function parameters were ln-transformed. Models were adjusted for maternal week, age, ethnicity, BMI, education level, household smoking status, drinking status and occupation.

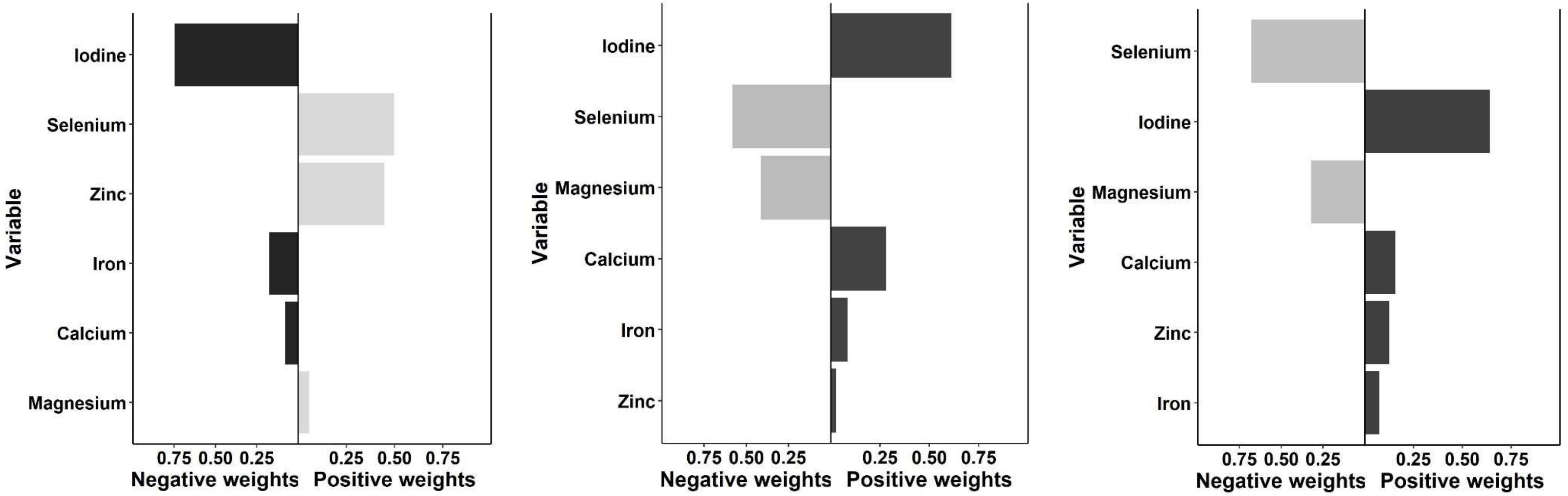

(A) (B) (C)

Figure S2 Contribution weight of each nutrient in overall effect on thyroid function parameters from quantile g-computation model. (A) (B) (C) represent the weights of positive or negative components of each mineral contributing to TSH, FT3 and FT4, respectively.

Table S2 Contribution proportions of each nutrient on thyroid function parameters in quantile g-computation.

Thyroid function parameters	Weights					
	Calcium	Magnesium	Iron	Zinc	Selenium	Iodine
TSH	-0.08	0.06	-0.17	0.45	0.50	-0.75
FT3	0.28	-0.42	0.08	0.03	-0.58	0.61
FT4	0.16	-0.32	0.07	0.13	-0.68	0.64

Note: TSH, thyroid stimulating hormone; FT3, free triiodothyronine; FT4, free thyroxine. All thyroid function parameters were ln-transformed. Models were adjusted for maternal week, age, ethnicity, BMI, education level, household smoking status, drinking status and occupation.

Table S3 Posterior inclusion probabilities (PIPs) of each nutrient in BKMR.

Thyroid function parameters	Weights					
	Calcium	Magnesium	Iron	Zinc	Selenium	Iodine
TSH	0.49	0.40	0.33	0.32	0.28	1.00
FT3	0.32	0.21	0.11	0.18	0.22	1.00
FT4	0.58	0.22	0.23	0.31	0.67	1.00

Note: TSH, thyroid stimulating hormone; FT3, free triiodothyronine; FT4, free thyroxine. All thyroid function parameters were ln-transformed. Models were adjusted for maternal week, age, ethnicity, BMI, education level, household smoking status, drinking status and occupation.

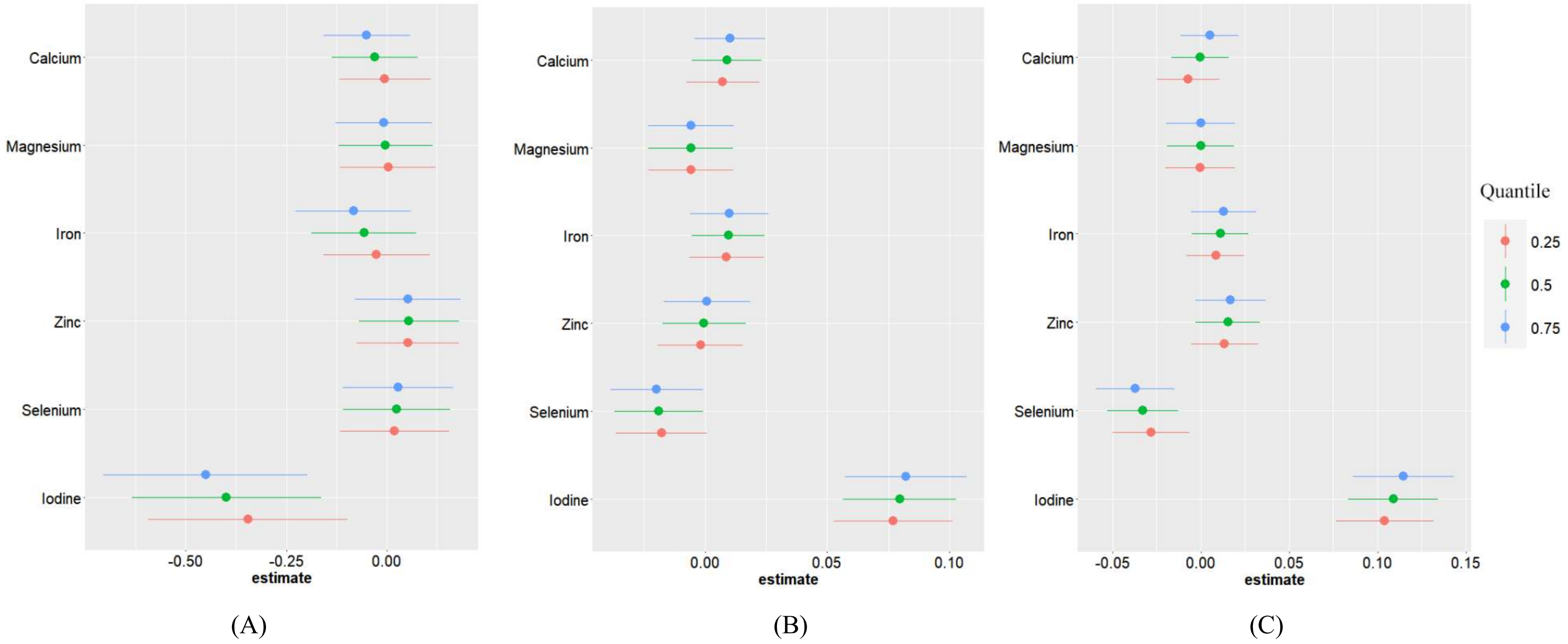

Figure S3 Single-exposure effects of each individual nutrient on thyroid hormone parameters from BKMR. It defined that the change in the (A) TSH (B) FT3 and (C) FT4 associated with a change in a particular nutrient from its 25th to its 75th percentile, where all of the other nutrients are fixed at a specific quantile (0.25, 0.50, or 0.75).

Table S4 Directions of associations between single nutrient, multi-nutrient mixtures and thyroid function parameters in three statistical methods

		Calcium	Magnesium	Iron	Zinc	Selenium	Iodine	Overall
TSH	GLM	↓	↓	↓	—	—	↓	
	g-comp	↓	↑	↓	↑	↑	↓	↓
	BKMR	↓	—	↓	↑	↑	↓	↓
FT3	GLM	↑	↑	↑	↑	—	↑	
	g-comp	↑	↓	↑	↑	↓	↑	↑
	BKMR	↑	↓	↑	—	↓	↑	↑
FT4	GLM	↑	—	↑	↑	—	↑	
	g-comp	↑	↓	↑	↑	↓	↑	↑
	BKMR	↑	—	↑	↑	↓	↑	↑
